# The General Public’s Awareness of Early Symptoms of and Emergency Responses to Acute Myocardial Infarction and Related Factors in South Korea: A National Public Telephone Survey

**DOI:** 10.2188/jea.JE20150074

**Published:** 2016-05-05

**Authors:** Hee-Sook Kim, HeyJean Lee, KeonYeop Kim, Hyeung-Keun Park, Ki-Soo Park, Gil Won Kang, Hee-Young Shin, Rock Bum Kim, Gyung-Jae Oh, Jae Hee Seo, Young-Hoon Lee

**Affiliations:** 1 Department of Epidemiology, Graduate School of Public Health, Seoul National University, Seoul, South Korea; 2 Division of Chronic Disease Control, Korea Centers for Disease Control and Prevention, Cheongju, South Korea; 3 Department of Preventive Medicine, Kangwon National University Hospital, Chuncheon, South Korea; 4 Regional Cardiocerebrovascular Disease Center, Kangwon National University Hospital, Chuncheon, South Korea; 5 Department of Preventive Medicine, Kyungpook National University School of Medicine, Daegu, South Korea; 6 Regional Cardiocerebrovascular Disease Center, Kyungpook National University Hospital, Daegu, South Korea; 7 Departments of Health Policy and Management, Medical School, Jeju National University, Jeju, South Korea; 8 Regional Cardiocerebrovascular Disease Center, Jeju National University Hospital, Jeju, South Korea; 9 Department of Preventive Medicine, Gyeongsang National University School of Medicine and Institute of Health Sciences, Jinju, South Korea; 10 Regional Cardiocerebrovascular Disease Center, Gyeongsang National University Hospital, Jinju, South Korea; 11 Department of Health Informatics & Management, College of Medicine, Chungbuk National University, Cheongju, South Korea; 12 Regional Cardiocerebrovascular Disease Center, Chungbuk National University Hospital, Cheongju, South Korea; 13 Department of Biomedical Science, Chonnam National University Medical School, Gwangju, South Korea; 14 Regional Cardiocerebrovascular Disease Center, Chonnam National University Hospital, Gwangju, South Korea; 15 Department of Preventive Medicine, Dong-A University College of Medicine, Busan, South Korea; 16 Regional Cardiocerebrovascular Disease Center, Dong-A University Hospital, Busan, South Korea; 17 Department of Preventive Medicine & Institute of Wonkwang Medical Science, Wonkwang University School of Medicine, Iksan, South Korea; 18 Regional Cardiocerebrovascular Center, Wonkwang University Hospital, Iksan, South Korea; 19 Regional Cardiocerebrovascular Disease Center, Chungnam National University Hospital, Daejeon, South Korea

**Keywords:** public, awareness, acute myocardial infarction, early symptoms, telephone survey

## Abstract

**Background:**

Prompt treatment affects prognosis and survival after acute myocardial infarction (AMI) onset. This study evaluated the awareness of early symptoms of AMI and knowledge of appropriate responses on symptom occurrence, along with related factors.

**Methods:**

Participants’ knowledge of the early symptoms of and responses to AMI onset were investigated using a random digit dialing survey. We included 9600 residents of 16 metropolitan cities and provinces in Korea.

**Results:**

The proportions of respondents who were aware of early symptoms of AMI ranged from 32.9% (arm or shoulder pain) to 79.1% (chest pain and discomfort). Of the respondents, 67.0% would call an ambulance if someone showed signs of AMI, 88.7% knew ≥1 symptom, 10.9% knew all five symptoms, and 3.1% had excellent knowledge (correct identification of all five AMI symptoms, not answering “Yes” to the trap question, and correctly identifying calling an ambulance as the appropriate response when someone is exhibiting AMI symptoms). The odds ratio (OR) for having excellent knowledge was significantly higher for those who graduated college or higher (OR 3.42; 95% confidence interval [CI], 1.09–10.76) than for those with less than a primary school education, as well as for subjects with AMI advertisement exposure (OR 1.49; 95% CI, 1.10–2.02) and with knowledge of AMI (OR 1.63; 95% CI, 1.16–2.27). The 60- to 79-year-old group had significantly lower OR for excellent knowledge than the 20- to 39-year-old group (OR 0.53; 95% CI, 0.28–0.99).

**Conclusions:**

Awareness of AMI symptoms and the appropriate action to take after symptom onset in South Korea was poor. Therefore, educational and promotional strategies to increase the overall awareness in the general public, especially in the elderly and those with low education levels, are needed.

## INTRODUCTION

Ischemic heart disease is the number one cause of death in the world, with approximately 7.4 million deaths in 2012 due to the disease.^1^ According to Korea’s Annual Report on the Cause of Death 2013,^2^ heart disease ranked third behind cancer and cerebrovascular diseases, with a mortality rate of 50.2 per 100 000 people, representing a 42% increase from the rate of 35.3 per 100 000 people 10 years earlier (in 2003). Owing to the rapidly aging population, the disease burden associated with heart diseases, including acute myocardial infarction (AMI), is expected to increase.

AMI, a form of ischemic heart disease, is an acute cardio-cerebrovascular disease in which prompt treatment after onset has unconditional effects on patient prognosis and mortality. Every 30-minute decrease in reperfusion time for AMI decreases the mortality rate by 1.5%^3^; when reperfusion occurs within 3 hours, the mortality rate improves by 23%, and when it occurs within 1 hour of symptom onset, the mortality rate improves by 50%.^4^ The key to early medical intervention is patient arrival at a medical facility immediately after the recognition of early symptoms. However, only 42.2% of patients in Korea arrive at the emergency room within the golden time (ie, 3 hours of occurrence).^5^ Delayed emergency room admission can be caused by delayed recognition of symptoms (especially in the elderly), nighttime onset, transfer from another hospital, and failure to utilize the emergency medical service (EMS). Among the delays that occur prior to hospital arrival, patient-related factors include not realizing the heart attack is occurring and an inadequate response even when the heart attack is recognized.^6^

A number of international studies have analyzed the general public’s knowledge of the early symptoms of and risk factors for AMI or AMI-related EMS calls,^7^^–^^9^ but there have been few similar studies conducted in Asia. The only awareness study conducted in Korea was limited to a specific region, targeted the elderly,^10^ and primarily focused on patients admitted for AMI.

The present study aimed to provide strategies to improve the awareness of the early symptoms of AMI by determining the current levels of knowledge of these symptoms and responses at the time of AMI onset in the general public from 16 metropolitan cities and provinces in South Korea, as well as factors related to the levels of knowledge.

## METHODS

### Participants

The target population comprised all adult men and women aged 19–79 years, based on Statistics Korea’s 2010 census (36 480 435 people). The survey population consisted of residents in 16 metropolitan cities and provinces in South Korea at the time of the survey, and the same theoretical considerations were used for the target population to identify one person from each household with a landline and those who used a mobile phone. From the survey population, the survey sample included 600 people from each city or province (total 9600 subjects); the sample was proportionally distributed by gender and age. The present study received a review exemption approval from the Institutional Review Board of Wonkwang University Hospital, which oversaw all of the study’s survey activities (WKUH 201410-HRE-068).

### Data collection

A structured questionnaire was used as the survey instrument. The section of the questionnaire on early symptoms of AMI was based on parts of the Behavioral Risk Factor Surveillance System,^11^ which is a state-level telephone health survey system in the United States. The participants were instructed as follows: “If you think the following sentence pertains to symptoms of acute myocardial infarction, please answer ‘Yes’; if you do not think so, answer ‘No’; if you are unsure, answer ‘I do not know’.” Symptoms included the following: sudden pain or discomfort in the jaw, neck, or back; sudden weakness or dizziness; sudden pain or discomfort in the chest; sudden blurred vision in one or both eyes (a trap question); sudden pain or discomfort in the arms or shoulders; and sudden shortness of breath. To evaluate the possibility that respondents may answer “Yes” to all the symptoms, a trap question about vision impairment was included. Unlike other symptom questions, where the correct answer is “Yes,” the correct answer for the trap question is “No.”

Additionally, to the question, “If someone shows symptoms of acute myocardial infarction, what do you think you should do first?”, the respondents chose one answer among “take them to a hospital”, “take them to an Oriental medicine hospital”, “call an ambulance”, “contact family”, “other actions”, or “do not know.”

The data also included respondents’ socio-demographic characteristics, including gender, education level, and monthly household income; past medical history of hypertension, diabetes, or dyslipidemia; history (diagnosis) of AMI in self, immediate family members, or acquaintances (including relatives) or neighbors; knowledge of AMI; being aware of the need for prompt treatment; and exposure to public service campaigns and/or advertisements.

The survey was conducted using a random digit dialing (RDD) method. At the time, to minimize selection bias, 20% of the total sample was surveyed via mobile phones to include people without landline telephones. Moreover, in consideration of different in-house rates for landline telephones, the time use survey from Statistics Korea was utilized for time-balanced quasi-quota sampling. The survey was conducted for 1 month, from October 15 to November 13, 2012. Analysis of the telephone survey showed a landline response rate of 20.3% and a cellular phone response rate of 15.6% (Figure). The response rate was calculated as: Response rate = 1 − {[(absent + busy line + rejected + interrupted)]/[(number of available phone numbers − not a subject − quota over − undependable)]}.

**Figure.**
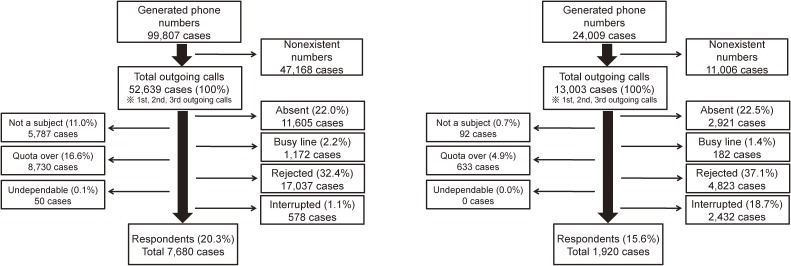
Respondent collection process through RDD phone survey (Left: landline telephone, Right: cellular phone).

### Statistical analysis

IBM SPSS Statistics for Windows version 20 (IBM Corp., Armonk, NY, USA) was used for data analysis. Chi-squared tests were performed to compare the differences in awareness of each symptom and appropriate responses based on socio-demographic characteristics, including gender, age, education level, and monthly household income, as well as risk factors, including family history of AMI and pre-existing conditions (hypertension, diabetes, or dyslipidemia). Logistic regression analysis was conducted to obtain the odds ratios (ORs) and 95% confidence intervals (CIs) for the factors related to excellent AMI knowledge (knowing all five symptoms of AMI, knowing that the one trap question was not a symptom of AMI, and identifying “calling an ambulance” as the appropriate response when someone is showing symptoms of AMI). The logistic regression analysis was performed after excluding subjects with direct (personal history of AMI) or indirect experience of AMI (diagnosis in immediate family members, other relatives, acquaintances, or neighbors). The Hosmer-Lemeshow test was used to test the model’s goodness-of-fit. The statistical significance level (α) was set at 0.05.

## RESULTS

A total of 9600 subjects (49.7% men and 50.3% women) with an average age of 45.1 (standard deviation, 15.3) years participated in the telephone survey. Table 1 shows the participants’ general characteristics. When the 16 metropolitan cities and provinces were grouped, with one group consisting of the capital areas (Seoul and Kyung Gi Province) and metropolitan cities and the other group consisting of all other provinces, each group included 50% of the total sample. In terms of the respondents’ medical history of pre-existing conditions for cardio-cerebrovascular diseases, the percentages for hypertension, diabetes, and dyslipidemia were 15.3%, 4.7%, and 7.5%, respectively, and a history of AMI was reported at rates of 1.4% for self, 8.8% for immediate family, and 15.7% for acquaintances (including relatives) or neighbors.

**Table 1.** General characteristics of respondents

Characteristics	*n*	%
Total	9600	100.0
Gender		
Male	4768	49.7
Female	4832	50.3
Age group, years		
20–39	3809	39.7
40–59	3907	40.7
60–79	1884	19.6
Education		
Less than primary school	902	9.4
Middle/high school	3955	41.2
College graduate or higher	4559	47.5
Nonresponse/refusal	184	1.9
Average monthly household income, 10 000 Won		
≤100	1199	12.5
101–300	3194	33.3
301–500	2654	27.6
>500	1421	14.8
Nonresponse/refusal	1132	11.8
Region of residence		
Capital area or metropolitan city	4800	50.0
Province	4800	50.0
Hypertension	1465	15.3
Diabetes mellitus	451	4.7
Dyslipidemia	717	7.5
History of AMI		
Respondent	137	1.4
Immediate family	840	8.8
Relative, acquaintance, or neighbor	1506	15.7
Exposure to AMI-related public service announcements or promotional materials	2566	26.7
Knowledge of AMI	6274	65.4
Being aware of the need for prompt treatment for AMI	8775	91.4

AMI, acute myocardial infarction.

Awareness of early symptoms of AMI and the appropriate action at the time of occurrence (ie, calling an ambulance) according to socio-demographic and risk factors are shown in Table 2. The proportion of subjects who were aware of major symptoms of AMI was highest for “pain or discomfort in the chest” (79.1%), followed by “difficulty in breathing” (70.2%); “weakness or dizziness” (49.4%); “pain or discomfort in the jaw, neck, or back” (34.8%); and “pain or discomfort in the arms or shoulders” (32.9%). Moreover, only 23.9% of the respondents answered the trap question (ie, the symptom of visual impairment) correctly by stating “No.” Of all respondents, 33.7% answered “Yes” to the trap question and 6.3% answered “Yes” to all five AMI symptoms and the trap question (data not shown). A total of 67.0% of the respondents answered that they would call an ambulance when someone showed symptoms of AMI. Generally, knowledge of symptoms and appropriate response were higher in those with a higher education level, those with a higher income level, and those who resided in the capital area or metropolitan cities than in those with a lower education level, those with a lower income level, and those who resided in provincial areas, respectively. Differences in the awareness of each symptom were also observed based on medical history, such as pre-existing conditions and prior disease history. Respondents without hypertension showed greater awareness of “pain or discomfort in the chest”; “pain or discomfort in the jaw, neck, or back”; “vision impairment”; and the appropriate response than those with hypertension. Respondents without diabetes were significantly more likely aware of “pain or discomfort in the chest”, “difficulty in breathing”, and “vision impairment”, as well as “calling an ambulance” (67.6% vs 54.5%), than those with diabetes. Awareness of “pain or discomfort in the chest” and “weakness or dizziness” was higher in those with dyslipidemia than in those without dyslipidemia.

**Table 2.** Awareness of each symptom of acute myocardial infarction and calling an ambulance

Characteristics	Percentage (%)						

	Pain or discomfort in the jaw, neck, or back	Weakness or dizziness	Pain or discomfort in the chest	Vision impairment (trap question)^a^	Pain or discomfort in the arms or shoulders	Difficulty breathing	Calling an ambulance
Total	34.8	49.4	79.1	23.9	32.9	70.2	67.0
Gender							
Male	35.2	47.0	78.0	24.9	31.1	67.5	67.9
Female	34.5	51.8	80.0	22.8	34.6	72.9	66.1
*P value*	*0.503*	*<0.001*	*0.016*	*0.019*	*<0.001*	*<0.001*	*0.062*
Age group, years							
20–39	36.2	46.7	77.8	27.4	35.7	66.4	71.5
40–59	36.8	55.0	86.4	23.9	33.3	77.0	70.2
60–79	28.1	43.3	66.3	16.6	26.5	63.6	51.5
*P value*	*<0.001*	*<0.001*	*<0.001*	*<0.001*	*<0.001*	*<0.001*	*<0.001*
Education							
Less than primary school	19.8	32.5	51.4	11.5	20.6	54.3	43.1
Middle/high school	32.8	49.4	79.0	24.6	31.0	70.9	66.3
College graduate or higher	39.9	53.2	84.9	25.8	37.0	73.1	72.9
Nonresponse/refusal	28.8	36.4	69.6	19.6	30.4	61.4	53.3
*P value*	*<0.001*	*<0.001*	*<0.001*	*<0.001*	*<0.001*	*<0.001*	*<0.001*
Average monthly household income, 10 000 Won							
≤100	24.4	37.9	58.8	16.6	23.9	59.4	49.7
101–300	34.8	48.7	81.3	25.6	33.0	71.7	69.9
301–500	37.6	54.7	85.9	24.8	33.7	74.8	72.3
>500	40.3	54.8	86.5	27.4	38.6	74.8	70.7
Nonresponse/refusal	32.6	44.3	68.7	20.1	33.1	61.0	60.1
*P value*	*<0.001*	*<0.001*	*<0.001*	*<0.001*	*<0.001*	*<0.001*	*<0.001*
Region of residence							
Capital area or metropolitan city	35.7	49.8	80.7	25.1	33.6	72.3	68.5
Province	34.0	49.0	77.4	22.7	32.2	68.1	65.6
*P value*	*0.083*	*0.414*	*<0.001*	*0.006*	*0.123*	*<0.001*	*0.003*
Hypertension							
Yes	32.3	48.3	76.0	21.4	31.1	70.4	61.3
No	35.3	49.6	79.6	24.3	33.2	70.2	68.0
*P value*	*0.026*	*0.354*	*0.002*	*0.015*	*0.102*	*0.872*	*<0.001*
Diabetes mellitus							
Yes	32.2	46.3	72.9	16.0	31.9	65.9	54.5
No	35.0	49.5	79.4	24.3	33.0	70.4	67.6
*P value*	*0.219*	*0.187*	*0.001*	*<0.001*	*0.651*	*0.039*	*<0.001*
Dyslipidemia							
Yes	31.4	54.3	84.7	24.4	30.0	73.1	69.7
No	35.1	49.0	78.6	23.8	33.1	70.0	66.8
*P value*	*0.043*	*0.007*	*<0.001*	*0.723*	*0.084*	*0.079*	*0.107*
History of AMI							
Respondent	51.8	66.4	89.8	23.4	43.8	81.8	60.6
Immediate family	39.3	57.3	87.1	26.3	39.2	78.2	66.8
No	34.1	48.3	78.1	23.6	32.1	69.2	67.1
*P value*	*<0.001*	*<0.001*	*<0.001*	*0.219*	*<0.001*	*<0.001*	*0.267*
History of AMI among relatives, acquaintances, or neighbors							
Yes	38.7	56.6	88.8	27.7	35.4	79.1	69.9
No	34.1	48.0	77.2	23.2	32.4	68.5	66.5
*P value*	*0.001*	*<0.001*	*<0.001*	*<0.001*	*0.025*	*<0.001*	*0.009*
Exposure to AMI-related public service announcements or promotional materials							
Yes	41.4	58.8	87.7	25.5	39.2	78.4	67.7
No	32.5	45.9	75.9	23.3	30.6	67.2	66.7
*P value*	*<0.001*	*<0.001*	*<0.001*	*0.024*	*<0.001*	*<0.001*	*0.364*
Knowledge of AMI							
Yes	40.8	57.3	88.9	25.9	36.9	77.8	69.9
No	23.7	34.4	60.5	20.1	25.3	55.9	61.5
*P value*	*<0.001*	*<0.001*	*<0.001*	*<0.001*	*<0.001*	*<0.001*	*<0.001*
Being aware of the need for prompt treatment for AMI							
Yes	36.2	51.4	81.9	24.6	34.0	72.7	68.7
No	20.0	28.2	49.1	16.4	21.1	43.4	49.0
*P value*	*<0.001*	*<0.001*	*<0.001*	*<0.001*	*<0.001*	*<0.001*	*<0.001*

AMI, acute myocardial infarction.

^a^Correct answer (answered ‘no’) percentage.

In terms of AMI history, those who had experienced an AMI in the past had higher awareness of each individual symptom, except for the trap question, than those who had not previously experienced an AMI. Respondents whose family members had been diagnosed with AMI also showed higher awareness of all five symptoms and the trap question, than those without diagnosed family members. However, the percentage of subjects who identified the appropriate responses decreased from those who had never been diagnosed (67.1%) to those who had family members diagnosed (66.8%) and those who had been diagnosed themselves (60.6%). Furthermore, those whose acquaintances (including relatives) or close neighbors were diagnosed with AMI, those who were exposed to advertisements about AMI (excluding calling an ambulance), those who were personally knowledgeable about AMI, and those who were aware that the disease requires prompt treatment had a higher level of knowledge regarding the five symptoms, trap question, and “calling an ambulance” than their counterparts.

Most (88.7%) of the respondents had knowledge of at least one symptom, while only 10.9% knew all five symptoms (Table 3). Only 4.5% of the participants had knowledge of all five symptoms as well as the trap question (for the 5 symptoms of AMI, a response of “Yes” was defined as being aware of the symptom; for the trap question, however, a response of “No” and “I do not know,” was defined as being aware of the trap question and this definition was also applied to the trap question for excellent knowledge), while 3.1% possessed excellent knowledge (five symptoms of AMI, trap question, calling an ambulance). A total of 7.2% had knowledge of all five symptoms of AMI as well as the importance of calling an ambulance but not the trap question (not noted in the table). In terms of socio-demographic characteristics, higher awareness of the symptoms was observed in women, the 40- to 50-year-old age group, those with a higher education level, those with a higher income level, and residents of the capital area or metropolitan cities (92.2% of respondents in Gyeonggi Province and 84.5% of respondents in South Jolla Province were aware of one or more symptom, and 13.0% of respondents in Daegu City and 9.5% of respondents in South Jolla Province were aware of all five symptoms). For pre-existing conditions, the respondents who did not have hypertension or diabetes had higher awareness for one, two, and three or more symptoms, while those with dyslipidemia showed higher awareness for one and two or more symptoms. As for history of AMI, those who had been previously diagnosed showed greater awareness than those who had not been diagnosed for one or more symptom (96.4% vs 87.8%), all five symptoms (24.8% vs 10.3%), five symptoms and the trap question (15.3% vs 4.1%), and excellent knowledge (13.1% vs 2.7%). The respondents who answered that they had been exposed to advertisements about AMI, those who were knowledgeable about AMI, and those who knew that the disease required prompt treatment had a better knowledge of all symptoms than those who had answered otherwise.

**Table 3.** Awareness of acute myocardial infarction symptoms by number of symptoms

Characteristics	Percentage (%)						

	≥1	≥2	≥3	≥4	5	6^a^	Excellent knowledge^b^
Total	88.7	78.9	57.5	30.5	10.9	4.5	3.1
Gender							
Male	88.7	77.6	54.5	28.1	9.8	4.2	2.9
Female	88.7	80.2	60.3	32.8	11.9	4.8	3.2
*P value*	*0.942*	*0.002*	*<0.001*	*<0.001*	*0.001*	*0.154*	*0.344*
Age group, years							
20–39	87.9	77.7	56.4	29.7	11.1	4.9	3.6
40–59	94.5	85.7	63.3	33.2	11.6	4.7	3.0
60–79	78.1	67.4	47.5	26.2	8.8	3.6	2.0
*P value*	*<0.001*	*<0.001*	*<0.001*	*<0.001*	*0.004*	*0.089*	*0.004*
Education							
Less than primary school	66.4	53.2	36.1	17.3	5.7	2.0	0.8
Middle/high school	89.6	79.1	56.6	28.6	9.3	3.9	2.5
College graduate or higher	92.8	84.4	62.9	34.9	13.2	5.6	4.0
Nonresponse/refusal	75.5	65.2	45.7	26.6	13.6	4.9	3.3
*P value*	*<0.001*	*<0.001*	*<0.001*	*<0.001*	*<0.001*	*<0.001*	*<0.001*
Average monthly household income, 10 000 Won							
≤100	72.6	61.5	42.3	21.3	6.7	2.5	1.6
101–300	91.5	81.2	57.7	29.1	10.0	4.1	2.8
301–500	93.6	85.2	63.0	33.7	11.2	5.0	3.7
>500	93.6	85.1	64.7	36.5	15.2	6.3	4.3
Nonresponse/refusal	80.0	68.6	50.6	28.9	11.5	4.7	2.7
*P value*	*<0.001*	*<0.001*	*<0.001*	*<0.001*	*<0.001*	*<0.001*	*<0.001*
Region of residence							
Capital area or metropolitan city	90.1	80.4	58.6	31.5	11.5	4.9	3.3
Province	87.3	77.5	56.3	29.4	10.2	4.2	2.9
*P value*	*<0.001*	*0.001*	*0.026*	*0.021*	*0.045*	*0.141*	*0.194*
Hypertension							
Yes	87.0	76.2	54.7	29.1	11.0	4.6	2.6
No	89.0	79.4	57.9	30.7	10.8	4.5	3.2
*P value*	*0.024*	*0.005*	*0.022*	*0.236*	*0.867*	*0.842*	*0.239*
Diabetes mellitus							
Yes	84.3	73.2	52.1	27.9	11.8	3.1	2.0
No	88.9	79.2	57.7	30.6	10.8	4.6	3.1
*P value*	*0.002*	*0.002*	*0.019*	*0.234*	*0.535*	*0.133*	*0.171*
Dyslipidemia							
Yes	91.8	84.2	57.6	30.0	9.8	4.3	2.9
No	88.4	78.5	57.4	30.5	11.0	4.6	3.1
*P value*	*0.007*	*<0.001*	*0.936*	*0.775*	*0.324*	*0.771*	*0.804*
History of AMI							
Respondent	96.4	89.8	75.2	47.4	24.8	15.3	13.1
Immediate family	96.1	87.3	65.8	37.0	14.9	7.0	5.1
No	87.8	77.9	56.4	29.5	10.3	4.1	2.7
*P value*	*<0.001*	*<0.001*	*<0.001*	*<0.001*	*<0.001*	*<0.001*	*<0.001*
History of AMI among relatives, acquaintances, or neighbors							
Yes	96.1	89.0	66.1	35.2	12.2	4.3	2.7
No	87.3	77.0	55.8	29.6	10.6	4.6	3.2
*P value*	*<0.001*	*<0.001*	*<0.001*	*<0.001*	*0.081*	*0.647*	*0.378*
Exposure to AMI-related public service announcements or promotional materials							
Yes	95.6	88.0	67.3	39.8	14.9	6.4	4.0
No	86.2	75.6	53.9	27.1	9.4	3.9	2.7
*P value*	*<0.001*	*<0.001*	*<0.001*	*<0.001*	*<0.001*	*<0.001*	*0.001*
Knowledge of AMI							
Yes	96.6	88.7	67.0	36.4	12.9	5.4	3.7
No	73.7	60.4	39.4	19.2	7.1	3.0	1.8
*P value*	*<0.001*	*<0.001*	*<0.001*	*<0.001*	*<0.001*	*<0.001*	*<0.001*
Being aware of the need for prompt treatment for AMI							
Yes	91.3	81.8	59.8	31.9	11.4	4.8	3.3
No	61.1	48.7	32.1	14.9	5.0	2.1	1.2
*P value*	*<0.001*	*<0.001*	*<0.001*	*<0.001*	*<0.001*	*<0.001*	*0.001*

AMI, acute myocardial infarction.

^a^Includes all five symptoms (a response of “Yes”) and the trap question (a response of “No” or “I do not know” to the trap question was counted).

^b^Includes five symptoms of AMI (a response of “Yes”), the trap question (a response of “No” or “I do not know” to the trap question was counted), and appropriate response (calling an ambulance).

Examination of the OR of excellent knowledge (Table 4) indicated that ORs increased with education level; a significantly higher OR was observed in those who graduated college or higher (OR 3.42; 95% CI, 1.09–10.76), compared to those with less than a primary school education. The high-risk group with pre-existing conditions, including hypertension or diabetes, had lower OR than the non-risk group, but this difference was not significant. Excellent knowledge was significantly and positively correlated with AMI advertisement exposure (OR 1.49; 95% CI, 1.10–2.02) and knowledge of AMI (OR 1.63; 95% CI, 1.16–2.27) compared to lack of AMI advertisement exposure or knowledge of AMI, respectively; a significant negative correlation was observed for those aged 60–70 years (OR 0.53; 95% CI, 0.28–0.99) compared to those aged 20–30 years.

**Table 4.** Multivariable logistic regression analysis^a^: factors related to excellent knowledge^b^

Parameter	Odds ratio	95% CI
Gender		
Male	1.000	
Female	1.123	0.848–1.486
Age group, years		
20–39	1.000	
40–59	0.734	0.536–1.003
60–79	**0.527**	0.282–0.985
Education		
Less than primary school	1.000	
Middle/high school	2.238	0.728–6.875
College graduate or higher	**3.420**	1.087–10.762
Nonresponse/refusal	3.365	0.775–14.607
Average monthly household income, 10 000 Won		
≤100	1.000	
101–300	0.927	0.442–1.945
301–500	0.922	0.431–1.972
>500	1.212	0.555–2.647
Nonresponse/refusal	0.994	0.447–2.212
Region of residence		
Province	1.000	
Capital area or metropolitan city	1.144	0.864–1.515
Hypertension		
No	1.000	
Yes	0.923	0.560–1.519
Diabetes mellitus		
No	1.000	
Yes	0.830	0.333–2.068
Dyslipidemia		
No	1.000	
Yes	1.057	0.591–1.891
Exposure to AMI-related public service announcements or promotional materials		
No	1.000	
Yes	**1.490**	1.100–2.018
Knowledge of AMI		
No	1.000	
Yes	**1.626**	1.163–2.274
Being aware of the need for prompt treatment for AMI		
No	1.000	
Yes	1.867	0.939–3.711

AMI, acute myocardial infarction; CI, confidence interval.

The analysis was performed excluding subjects with direct (personal history of AMI) or indirect experience of AMI (diagnosis in family members or relatives or neighbors).

^a^The *P* value of the Hosmer-Lemeshow goodness-of-fit tests was >0.05.

^b^Includes five symptoms of AMI (a response of “Yes”), the trap question (a response of “No” or “I do not know” to the trap question was counted), and appropriate response (calling an ambulance).

## DISCUSSION

The total time from symptom onset to treatment includes delays prior to hospital arrival (ie, time elapsed in deciding what treatment to seek and transport time to a hospital with treatment capabilities) and delays at the hospital (ie, time elapsed from arrival at the ER to treatment or surgery).^8^ Among these delays, the time for the patient to make a decision accounts for a significant portion of the delays prior to hospital arrival^12^; therefore, the present study surveyed and analyzed knowledge of early symptoms of and appropriate responses to AMI in the general public.

Compared with other countries, a very low percentage of the general public in South Korea is aware of each individual symptom as well as all of the symptoms of AMI collectively. Moreover, levels of awareness of the need for “calling an ambulance,” which is an appropriate response at the time of AMI symptom occurrence, and of related excellent knowledge are also lower than in other countries. The most common AMI symptom identified by respondents was “pain or discomfort in the chest” (79.1%), and the least commonly identified symptom was “pain or discomfort in the arms or shoulders” (32.9%). A study from Poland^7^ reported that 90% and 27% of the respondents knew symptoms of chest pain and arm or shoulder pain, respectively; a study from the United States^8^ reported that 92.0% and 49.3% were aware of symptoms of chest pain and jaw, neck, or back pain, respectively. Despite chest pain being the most typical symptom, awareness of this symptom in the present sample was lower than in other countries, and the symptom with the lowest level of awareness in the present study was also identified less than in the United States. Although direct comparison is difficult due to differences in some of the offered options^7^ or the question^8^ from previous studies, the rate of knowing the appropriate response to AMI symptoms (calling an ambulance) was also lower in Korea (67.0%) than in Poland (87.4%) and the United States (86.8%, for “dial 911 if someone is having a heart attack or stroke”). These trends are also apparent for the number of symptoms identified, for which the United States shows higher awareness than Korea for one or more of the five symptoms (96.9% vs 88.7%) and for all five symptoms (32.4% vs 10.9%). Moreover, the percentage of respondents having excellent knowledge is also lower in Korea (3.1%) than in the United States (10.7%). In contrast to our study, where responses of “No” and “I do not know” defined being aware of the trap question, the United States study used only “No” responses to define an accurate response to the trap question; therefore, the difference is even greater. Aside from the trap question, the proportions of respondents with knowledge of the five symptoms of AMI and knowing to call an ambulance were also notably different between Korea (7.2%) and the United States (30.6%).^13^ As such, effort should be taken in Korea to raise the level of awareness for not only the most typical symptom of “pain or discomfort in the chest” but also for other symptoms. According to several studies, approximately one-third of patients with a definitive diagnosis of myocardial infarction do not show chest pain at the time of hospital admittance,^14^^,^^15^ and 87% of patients with AMI experience one or more symptoms.^16^

The results of the present study suggest that a focused intervention for the group at high risk of cardio-cerebrovascular diseases (eg, those with pre-existing conditions) should be considered for future education and advertisement strategies. Although there were minor differences based on each of the pre-existing conditions, respondents with hypertension, diabetes, or dyslipidemia showed no differences in the awareness of early symptoms and the appropriate response compared with those without pre-existing conditions, and, in fact, the non-risk group showed slightly higher levels of awareness. These results contradict those of an overseas study,^13^ in which patients with pre-existing conditions (hypertension or dyslipidemia) demonstrated higher levels of awareness of early symptoms than the non-risk group.^13^ The proportion of subjects in the present study with excellent knowledge among subjects with personal history of AMI was lower than that in the United States, although a less rigid standard defining such knowledge was applied, compared to the United States (13.1% vs 16%). Those with pre-existing cardio-cerebrovascular conditions or a history of AMI are considered at high risk of AMI, and excellent knowledge of AMI symptoms is particularly important for these subjects. This is particularly true given that, within 6 years of an AMI, 18% of men and 35% of women experience a recurrence of AMI,^17^ and the second occurrence can feature symptoms that are different from the first.^18^

The univariate analyses indicated that the pattern of inequalities in awareness based on gender, age, education level, and monthly household income was similar to that in other countries.^8^^,^^13^ There were major differences in the awareness of individual symptoms between the major metropolitan areas and other regions, while similarities existed in the proportion of subjects with knowledge to call an ambulance (more than 10%) and the number of symptoms identified. These results should be considered in the development of future strategies to improve awareness.

Meanwhile, according to the multiple logistic regression analysis results, the lower rates of awareness in the middle-aged (40–59 years old) and elderly (60–79 years old) respondents compared with those in their 20s and 30s could be concerning, although the difference between middle-aged and younger respondents was not significant. AMI becomes more prevalent in men from the age of 40 years and occurs most often in those aged 50–59 years (29.2% of all cases).^19^ Furthermore, the different trends in awareness based on gender and education level in the present study supported the results of other studies^8^^,^^13^; however, the differences by gender were not statistically significant, which might be explained by the very low awareness of the trap question.

Awareness of the disease itself is related to knowledge of the symptomatic causes; considering that knowledge that the various symptoms are related to the heart reduces the delays prior to hospital arrival,^20^ improvements in the poor knowledge of AMI observed in the present study are urgently required. After adjusting for sociodemographic variables (gender, age, education level, income, and region) and disease history (hypertension, diabetes, and dyslipidemia), public relations (exposure to AMI-related public service announcements or promotional materials) was still an independent factor associated with excellent knowledge of AMI symptoms.

Overall, the level of awareness in Korea of the early symptoms of AMI is quantitatively and qualitatively lower than in other countries, including the United States. This can be attributed, in part, to a lower disease burden in Korea and therefore less awareness of this disease compared with other diseases, such as cancer or stroke. Heart disease has long been the leading cause of death in the United States, and the burden associated with heart disease has also been significant^21^; moreover, because care for this condition has been a high priority, there have been many related studies, in addition to educational and advertisement campaigns via mass media. The risk of and burden associated with heart diseases, including ischemic heart disease,^2^^,^^19^ is increasing in Korea, indicating that heart disease is an area that requires investment of considerable resources for improvements in care.

There are several strengths to this study. To the best of our knowledge, this is the first large-scale study conducted in Korea that used the general public as the sample to examine the awareness of early symptoms of AMI. Regarding methodology, the study strengths include the inclusion of respondents using mobile phones as well as landline telephones and a survey that reflected the time use survey in Korea. However, this study also has certain limitations, including bias related to the survey process, which cannot be disregarded. First, individuals of low socioeconomic status generally do not own landline telephones or mobile phones and may have been underrepresented in the telephone survey. In addition, the use of closed-ended questions during the telephone survey may have overestimated the level of awareness of the general public. Second, because the response rate is not high (landline telephone response rate, 20.3%; cellular phone response rate, 15.6%), one must be cautious in generalizing the results of this study. Third, because sampling in this study was not proportional to the population of each region, it does not represent the entire population of Korea, despite being a large sample. Fourth, 10.9% of respondents answered “Yes” to all 5 symptoms of AMI. In this survey, we used a trap question to assess whether respondents were simply answering “Yes” to all queries; 6.3% of respondents answered “Yes” to all 6 symptoms, including the trap question, so only 4.6% of respondents correctly answered all 6 symptoms, including a negative answer to the trap question (“No” + “I do not know”). This limitation is partly attributable to the questionnaire, which presented all awareness items in a series of closed questions. Assessment of the awareness of AMI symptoms should take into consideration the proportions of respondents who did not correctly answer the trap question. In this survey, 33.7%, 23.9%, and 42.4% of respondents answered “Yes”, “No”, and “I do not know”, respectively, to the trap question.

In conclusion, awareness of AMI symptoms and knowledge of the appropriate action to take upon symptom onset in South Korea was poor. Educational and promotional strategies to increase the overall awareness of AMI symptoms in the general public, especially in the elderly and those with low education levels, are needed. Future national or community surveillance systems should include indices related to knowledge regarding the awareness of early symptoms and responses, such as ‘awareness of signs and symptoms of AMI’ and ‘call 119 (emergency telephone number in Korea) after the onset of AMI symptoms’, to meet the needs for trend analysis and continued monitoring using data collected on a regular basis.
